# Network diffusion modeling predicts neurodegeneration in traumatic brain injury

**DOI:** 10.1002/acn3.50984

**Published:** 2020-02-27

**Authors:** Govinda R. Poudel, Juan F. Dominguez D, Helena Verhelst, Catharine Vander Linden, Karel Deblaere, Derek K. Jones, Ester Cerin, Guy Vingerhoets, Karen Caeyenberghs

**Affiliations:** ^1^ Mary MacKillop Institute for Health Research Australian Catholic University Melbourne VIC Australia; ^2^ Cognitive Neuroscience Unit School of Psychology Deakin University Burwood VIC Australia; ^3^ Department of Experimental Psychology Faculty of Psychology and Educational Sciences Ghent University Ghent Oost‐Vlaanderen Belgium; ^4^ Child Rehabilitation Centre Ghent University Hospital Ghent Oost‐Vlaanderen Belgium; ^5^ Department of Neuroradiology Ghent University Hospital Ghent Oost‐Vlaanderen Belgium; ^6^ Cardiff University Brain Research Imaging Centre (CUBRIC) School of Psychology Cardiff University Cardiff Wales United Kingdom

## Abstract

**Objective:**

Traumatic brain injury (TBI) is a heterogeneous disease with multiple neurological deficits that evolve over time. It is also associated with an increased incidence of neurodegenerative diseases. Accordingly, clinicians need better tools to predict a patient’s long‐term prognosis.

**Methods:**

Diffusion‐weighted and anatomical MRI data were collected from 17 adolescents (mean age = 15y8mo) with moderate‐to‐severe TBI and 19 healthy controls. Using a network diffusion model (NDM), we examined the effect of progressive deafferentation and gray matter thinning in young TBI patients. Moreover, using a novel automated inference method, we identified several injury epicenters in order to determine the neural degenerative patterns in each TBI patient.

**Results:**

We were able to identify the subject‐specific patterns of degeneration in each patient. In particular, the hippocampus, temporal cortices, and striatum were frequently found to be the epicenters of degeneration across the TBI patients. Orthogonal transformation of the predicted degeneration, using principal component analysis, identified distinct spatial components in the temporal–hippocampal network and the cortico‐striatal network, confirming the vulnerability of these networks to injury. The NDM model, best predictive of the degeneration, was significantly correlated with time since injury, indicating that NDM can potentially capture the pathological progression in the chronic phase of TBI.

**Interpretation:**

These findings suggest that network spread may help explain patterns of distant gray matter thinning, which would be consistent with Wallerian degeneration of the white matter connections (i.e., “diaschisis”) from diffuse axonal injuries and multifocal contusive injuries, and the neurodegenerative patterns of abnormal protein aggregation and transmission, which are hallmarks of brain changes in TBI. NDM approaches could provide highly subject‐specific biomarkers relevant for disease monitoring and personalized therapies in TBI.

## Introduction

The risk of neurodegenerative diseases (e.g., Parkinson’s disease, Alzheimer’s disease) is increased when traumatic brain injury (TBI) is sustained at an early age.^1^, ^2^ This observation is of particular concern, given the high annual incidence rates of childhood brain injuries (765 per 100.000 population) resulting from motor vehicle accidents, falls, sports, and abuse.^3^ Following the initial impact, the brain undergoes a series of gradual changes that often lead to more damage than the primary injuries.^4^ Among these secondary mechanisms, spread of pathology via the brain’s white matter network is believed to play an important role in the pathogenesis of TBI.^5^

This view is supported by recent studies using graph theoretical analyses demonstrating alterations in network measures, such as global efficiency, clustering coefficient, and betweenness‐centrality in TBI patients compared to healthy controls.^6^, ^7^ However, these changes in network metrics are unable to determine the patterns of degeneration within the brain networks.^8^ It is essential to understand how the initial brain trauma relates to future patterns of degeneration in TBI patients. Achieving this understanding will lead to more appropriate head injury management and reduce the risk of TBI‐initiated neurodegenerative diseases.

The present study employed a model of spread of pathology via brain networks based on the network diffusion model (NDM).^8^, ^12^ A growing number of studies have utilized NDM as a means to model the progression of neurodegenerative pathology on brain networks.^9^, ^10^, ^11^, ^12^, ^13^, ^14^, ^15^ Using longitudinal data from the Alzheimer’s Disease Neuroimaging Initiative (ADNI) database, Torok and colleagues (2018)^15^ showed that NDM and an inference optimization algorithm can successfully infer the regions of disease initiation (referred as seed regions) from which Alzheimer’s disease or Mild Cognitive Impairment tau pathology most likely originated. Our recent study^12^ demonstrated that the spread of mutant huntingtin protein, via the human brain connectome, accurately predicted the cortico‐striatal spatial pattern of degeneration in patients with Huntington’s disease. The NDM framework was also recently used for capturing the spatiotemporal progression of Parkinson’s disease.^9^, ^11^ These studies revealed that the substantia nigra was the most likely seed region, highlighting its role as one of the most atrophied and Lewy‐body‐rich regions in Parkinson’s disease. Another interesting finding was that the temporal sequencing of the regions predicted by the NDM was in close correspondence with the Braak’s Lewy‐body‐based staging scheme. The topography of neurodegenerative diseases is therefore well characterized by hallmark misfolded proteins, and NDM has been shown to successfully model their spread.

In this paper, we propose to extend NDM to understand the long‐term course of TBI. NDM applies to any first‐order diffusive process on a graph. Therefore, NDM may also be able to model the effect of progressive deafferentation and atrophy resulting from a traumatic brain injury (likely driven either by Wallerian degeneration of the white matter connections or, similar to the neurodegenerative conditions, via abnormal protein aggregation and transmission as suggested by recent findings).^16^, ^17^ In the present study, we used NDM to achieve precisely this in a cohort of TBI patients. Moreover, we implemented a novel automated inference method to identify several injury epicenters from which neurodegenerative pathology most likely originates in each individual patient. In addition, we employed principal component analysis to identify common neurodegenerative patterns predicted by diffusive processes across patients. Finally, we conducted exploratory correlation analyses to examine whether patterns of degenerative changes are associated with clinical measures.

## Materials and Methods

### Participants

In all, 36 children (17 TBI patients and 19 healthy controls, see Data S1 for demographic data) were recruited for the present study, which was part of a larger‐scale cognitive training study in pediatric TBI.^18^ Inclusion criteria for patients were as follows: (1) Age at injury: 10–17 years; (2) Injuries classified as moderate to severe using the Mayo Classification System^19^; and (3) In the chronic stage of injury at the time of assessment (1–5 years post injury).^20^ In total, 19 typically developing children were recruited via social networks of researchers to obtain gender‐ and age‐matched (maximum of ±6 months) controls for each TBI patient.

### Standard protocol approvals, registrations, and patient consents

The study was approved by the Ethics Committee of the Ghent University Hospital (#2014/0540) and written informed consent was obtained from both parents and participants in accordance with the Declaration of Helsinki.

### MRI acquisition

Anatomical scans were collected using a MPRAGE sequence^21^ (TR/TE = 2250/4.18 msec; TA = 5:14 min; flip angle = 9°; FOV = 256 mm; voxel size = 1.0 mm isotropic; slab thickness = 176 mm; BW = 150 Hz/pixel) and High Angular Resolution Diffusion Imaging (HARDI) scans^22^ consisting of a twice‐refocused spin echo sequence^23^ (60 contiguous transversal slices, FOV = 240 mm; voxel size = 2.5 mm isotropic, TR/TE = 10,800/83 msec, 64 noncollinear directions, b value = 1200 s/mm^2^, 1 b0, TA = 12:36 min) on a Siemens 3T TrioTim MRI scanner equipped with a 32‐channel head coil at Ghent University Hospital, Belgium.

### Connectome reconstruction

Cortical reconstruction and volumetric segmentation were performed with the Freesurfer image analysis suite (http://surfer.nmr.mgh.harvard.edu/). The details of the Freesurfer analysis of the same cohort are described in prior publications by Vander Linden et al. (2019a,b).^24^, ^25^ Briefly, this processing includes motion correction, removal of non‐brain tissue using a hybrid watershed/surface deformation procedure, automated Talairach transformation, segmentation of the subcortical white matter and deep gray matter volumetric structures, intensity normalization, tessellation of the gray matter white matter boundary, automated topology correction, and surface deformation following intensity gradients to optimally place the gray/white and gray/cerebrospinal fluid borders at the location where the greatest shift in intensity defines the transition to the other tissue class. Once these cortical models were complete, a parcellation of the cerebral cortex into units with respect to gyral and sulcal structure was performed. In the present study, a total of 82 gray matter brain regions were parcellated using the Desikan‐Killiany atlas.^26^ Freesurfer morphometric procedures have been demonstrated to show good test–retest reliability across scanner manufacturers and across field strengths. Quality assurance of the registration and segmentation was undertaken by visual inspection. In case of inaccuracies, manual editing was performed either by adding control points to help FreeSurfer identify the WM voxels or by removing the skull and dura in case they were considered to be parts of the brain.

Whole brain white matter networks were extracted from the HARDI scans, using previously described methodology.^27^ Raw diffusion‐weighted images were corrected for eddy current, motion, and B1‐field inhomogeneity using Mrtrix3. The anatomical T1‐weighted images were linearly registered to diffusion space using FSL. Constrained spherical deconvolution followed by second‐order integration over fiber orientation distributions (iFOD2) algorithm^28^ was used to reconstruct the tractograms. Spherical‐deconvolution informed filtering of tractograms (SIFT) was implemented to decrease reconstruction biases and improve biological plausibility.^29^

For each subject, the whole brain tractography and T1‐based parcellations were combined. The nodes were represented by 82 distinct regions, and for each possible node pair, interregional connectivity was defined as the number of reconstructed streamlines (NOS), representing the edges of the connectome. This resulted in a weighted adjacency matrix for each subject. Finally, the healthy brain connectome was derived by taking the average of all individual 19 82 × 82 control subjects' connectomes to form a single 82 × 82 control white matter connectome.

### Modeling network diffusion on the human brain connectome

We used Raj et al.’s (2012) network diffusion model (NDM), allowing progressive degenerative changes in TBI to be modeled as passive diffusion. The human brain connectome can be represented as a graph **G** = (**v**, **ε**), in which the passive diffusion model treats the edge **ε** as a conduit of spread in nodes **v**, such that network spread of pathology at time *t* can be modeled as:

(1) $$f\left(t\right)=e^{-\alpha H}f\left(0\right)$$

where *f*(*t*) denotes the vector characterizing the volumetric loss at node v*_i_* at time *t*, starting from an initial distribution given by *f*(0) at time zero. **H** is the graph Laplacian (defined as the difference between the degree matrix and adjacency matrix). Alpha (*α*) is the diffusion coefficient.

### A method to identify injury epicenters in each individual with TBI

**Figure 1:**
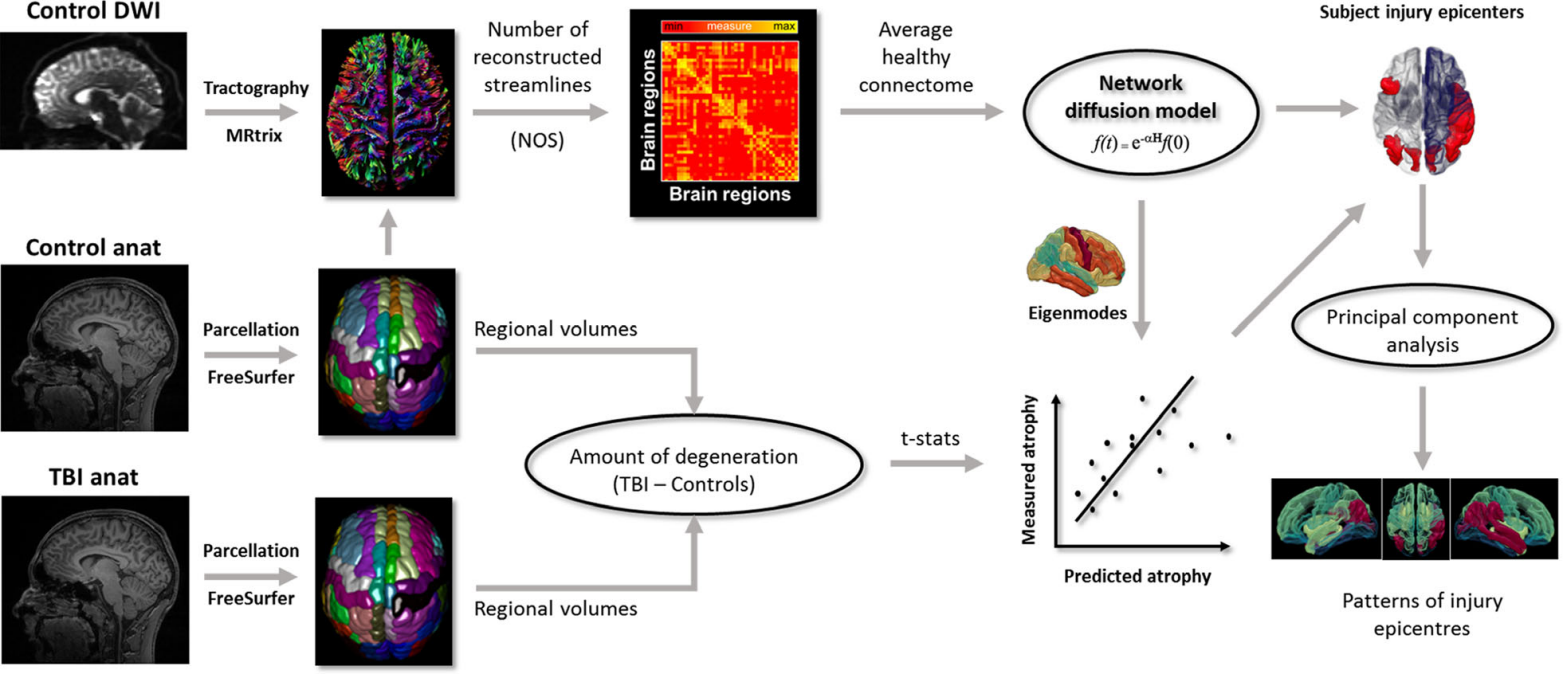
Overview of the workflow.

We applied NDM on the healthy human brain connectome to simulate the effect of spread of pathology in TBI (Fig. 1). This process was used to identify the brain regions (here also referred to as injury epicenters) from where diffusion seeding maximally predicted the neurodegeneration in each TBI individual. The following steps describe the process in greater detail:

1. Atrophy (i.e., relative volume loss compared to controls) in each patient was measured using *z*‐scores. A *z*‐score was computed as (*X* − *μ*)/*σ*, where *X* is the volume of the Desikan‐Killiany region in a TBI patient, and *μ* and *σ* are the mean and standard deviation of the volume of the same region in healthy controls, respectively. These *z*‐scores represent the current state of measured degeneration in each TBI patient.
2. The NDM was simulated on the healthy brain connectome. For each Desikan‐Killiany region, i (and initial condition *f* (0) = 1), the NDM (as per eq. 1) was run treating the region as a “seed” node to estimate the amount of diffusion from that seed to the 82 regions. This was done for 20 sequential time points, *t* = 0 to 19. The diffusion coefficient, *α*, was set to 0.25, as the TBI cohort were within 5 years since injury. This process generated an 82 × 20 matrix for each seed node, encapsulating diffusion of pathology (predicted atrophy) in 82 regions over time.
3. The measured atrophy in a given patient was correlated with atrophy predicted by NDM (from step 2). This generated a vector comprised of 82 correlation coefficient values for each time point, resulting in an 82 × 20 matrix. The highest correlation across the 20 time points for each seed region was determined and represented as vector R*_i_*. R*_i_* was set to 0 for all regions *i* for which *t_i_* = 0 or *t_i_* = 19. Furthermore, R*_i_* was also set to 0, if R*_i_* < 0 and R*_i_* < median value of R*_i_*. Finally, R*_i_* > 0 was set to 1, resulting in the initial configuration of injury epicenters. Next, R*_i_* was set as initial condition (*fo*) in the NDM, and the time point at which the correlation between measured and predicted atrophy was the highest was determined, such that *t_max_ = arg_maxt_ R *(f_t_*, y) where *R *(f_t_*, y) is a vector of correlations between measured (y) and predicted atrophy (*f_t_*). To avoid spurious correlation driven by seed regions, the data points corresponding to the seed regions were excluded when running the correlations between measured and predicted atrophy. This ensured that inferred seeds were not merely replicating the most atrophied regions and NDM offered predictive power above the correlation driven by the seed pattern alone.
4. We then used an algorithm (pseudocode provided in Supplemental Material) to identify the combination of seeds which achieve the highest correlation with the measured atrophy, using the initial condition (*R*_i_ and *t*_max_). Hence, the unique combination of seeds achieving the highest correlation between the measured and predicted degeneration was identified to be putative epicenters of injury. “Of note, early work used linear correlation between predicted atrophy and measured atrophy to identify the seed regions.”^10^, ^13^, ^14^ More recent work^15^ used *L1‐penalized optimization algorithm* in subject‐level analysis to identify seed vectors in each individual subject. Our approach has some similarities with the recent work by Torok et al. (2018)^15^ in that it identifies the initial guess seed regions using similar heuristics. However, for identifying optimal seed vector, we use a simple iterative combination technique with a focus on the combination of seeds that can achieve the best solution from a finite set of regions. We chose to use this algorithm for its computational simplicity, ability to identify combination of multiple seeds, and prioritize the seeds of higher predictive value. This approach is more suitable for a clinical population with multifocal contusive injuries. However, a limitation with our approach is the risk of overfitting and the availability of large solution space which can potentially result in a large seed vector. Further studies should explore other possibilities in the validation of our inference method.

### Principal component analysis

Principal component analysis (PCA) was conducted as an exploratory data analysis to identify common orthogonal patterns on NDM predicted atrophy maps across TBI patients. Specifically, PCA was used to identify a reduced set of spatial maps that contained most of the information in the predicted atrophy matrix. PCA was implemented using the “pca” command available in the Statistics and Machine Learning Toolbox (Matlab). The eigenvectors and eigenvalues of the mean‐centered input data covariance matrix were calculated via singular value decomposition. The relative size of each eigenvalue quantifies the total variance captured by that component, with the first principal component accounting for the most variance, and each subsequent component, progressively less. The anatomical maps corresponding to the first five components, explaining at least 60% of the variance, were visualized.

### Correlation analyses

Coefficients of determination (square of the maximum correlation value) between predicted and measured atrophy were correlated against the time since injury in TBI, after controlling for the effect of age. Also, the ability of model’s peak time to predict individual time since injury was investigated using correlation analysis. A significance level of 0.05 was adopted.

## Results

### Epicenters of injury in TBI inferred using network diffusion

**Figure 2:**
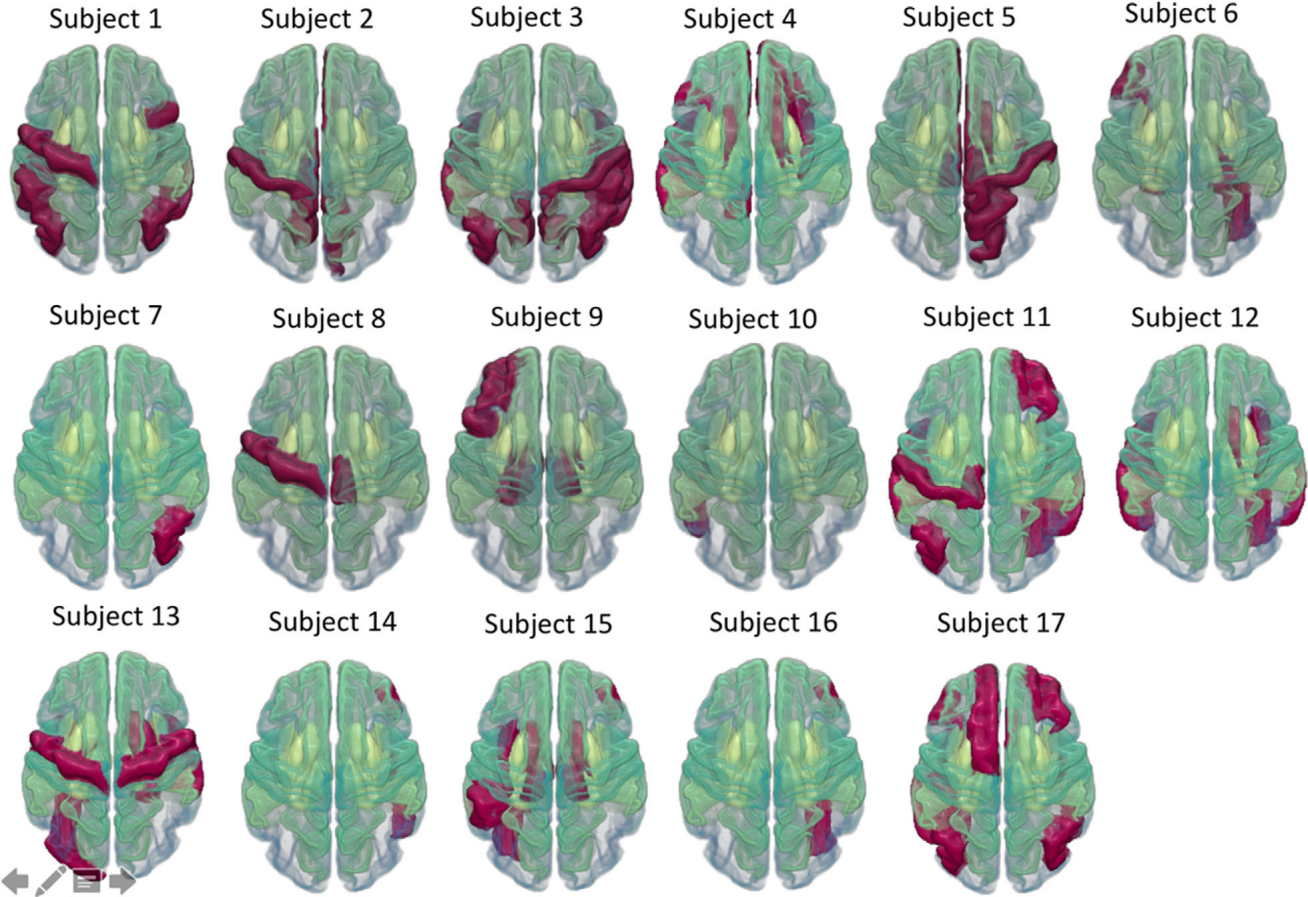
Visual representation of injury epicenters in 17 TBI individuals, mapped on the Desikan‐Killiany atlas (available in FreeSurfer). The red regions correspond to the brain regions within the injury epicenters. Modeling the network diffusion from these seeds achieved the highest correlation between measured and predicted atrophy.

Figure 2 shows the spatial location of NDM inferred injury epicenters in each individual. Both numbers and anatomical distribution of the inferred epicenters were highly heterogeneous across TBI subjects. For example, the injury epicenter in subjects 7 and 10 were localized to a single region in the right inferior parietal and left inferior temporal cortex, respectively. In contrast, injury epicenters in subjects 13, 15, and 17 comprised more than 10 regions distributed throughout the temporal, parietal, and frontal cortices and the striatum. The most prevalent brain regions within the inferred injury epicenters were located within the vicinity of the temporal cortex and the striatum. The list of brain regions within the inferred injury epicenters in all TBI patients is provided in Data S2.

**Figure 3:**
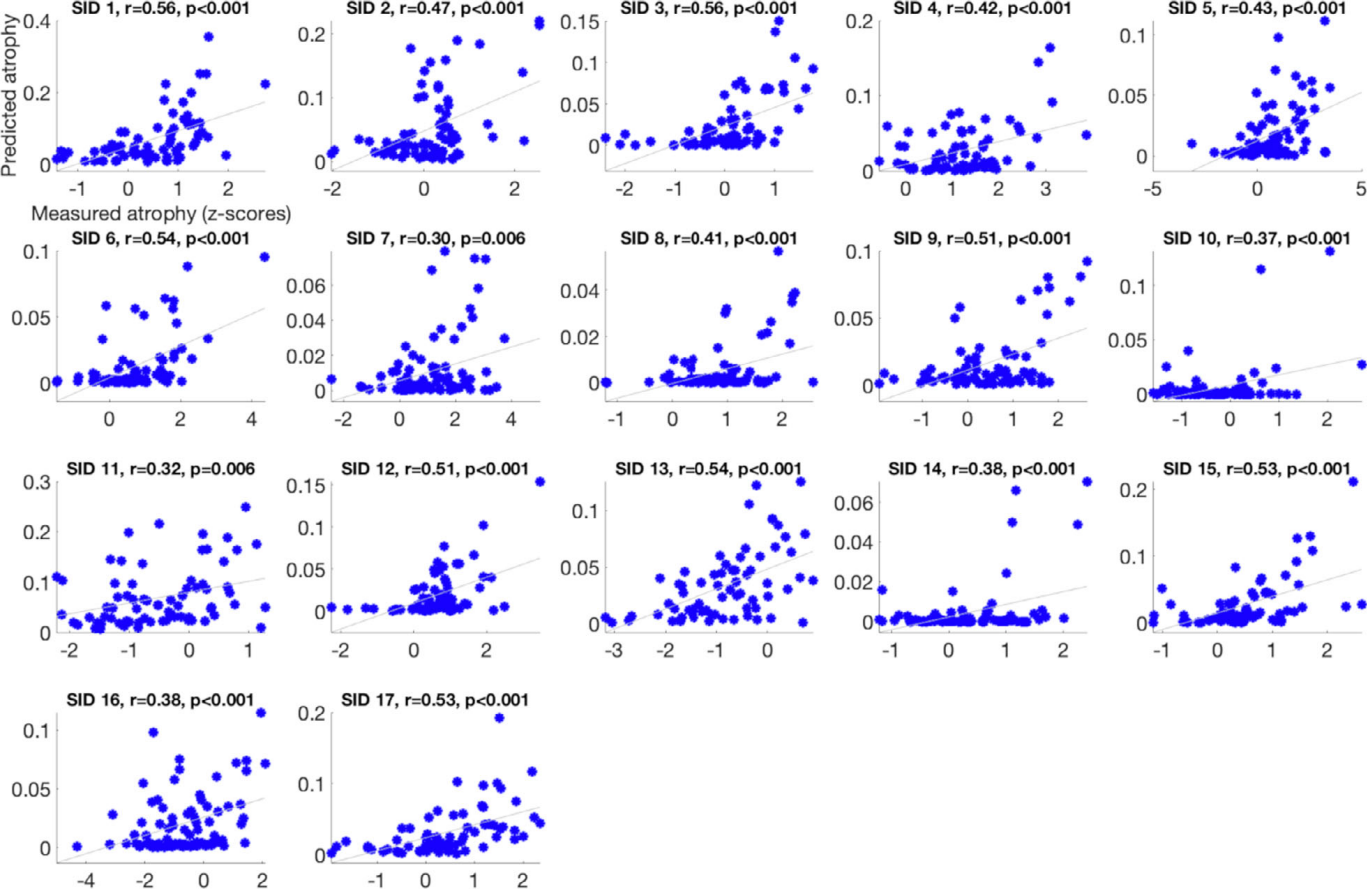
Scatterplots showing a linear association between predicted and measured atrophy in 17 subjects. Subject‐specific (represented by Subject ID (SID)) Pearson correlation coefficient values (*R*) and associated *P* values are provided within each scatterplot.

Figure 3 depicts the scatterplot of associations between the predicted and the measured atrophy. We observed significant positive moderate‐to‐strong correlations between the predicted and measured atrophy in all TBI individuals. However, the *R*‐coefficient values were highly variable across individuals (mean = 0.46, SD = 0.08, range = 0.30–0.56).

### Principal modes of atrophy maps

**Figure 4:**
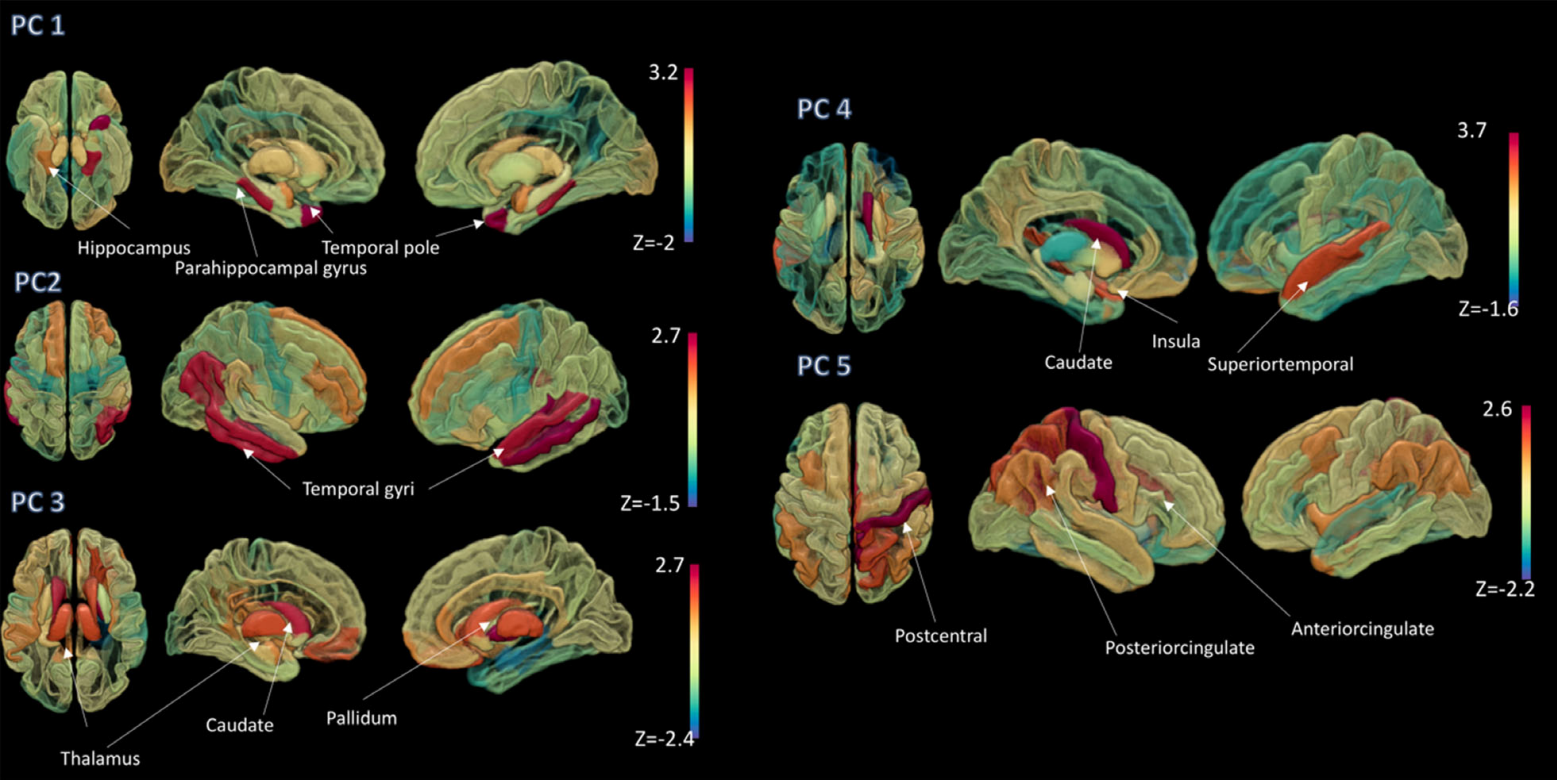
Patterns of injury epicenters. The first five principal components, explaining 68% of the variance, in the atrophy maps predicted by network diffusion modeling. Spatial maps corresponding to the coefficient of the corresponding eigenvectors (first to fifth), sorted from top (first eigenvector) to bottom (fifth eigenvector) are overlaid on a surface brain.

PCA revealed five components that accounted for 68% of the variance in the data (Fig. 4). Loadings on these five components were associated with distinct anatomical maps. The primary component, explaining 18% of the variance, had the highest loadings for the (para)hippocampal cortices and adjacent temporal pole. The second component (explaining 16% variance) was associated with the bilateral temporal cortices. The third component (13% variance) was related to the striatum, with the maximum loadings found for the caudate, pallidum, and thalamus. The fourth and fifth components (explaining ~10% variance) consisted of the caudate, insula, and superior temporal cortex (4th), and postcentral, posterior cingulate, and anterior cingulate gyri (5th). The remaining 12 components only explained small proportions of the total variance (<10%).

### Correlation analyses

**Figure 5:**
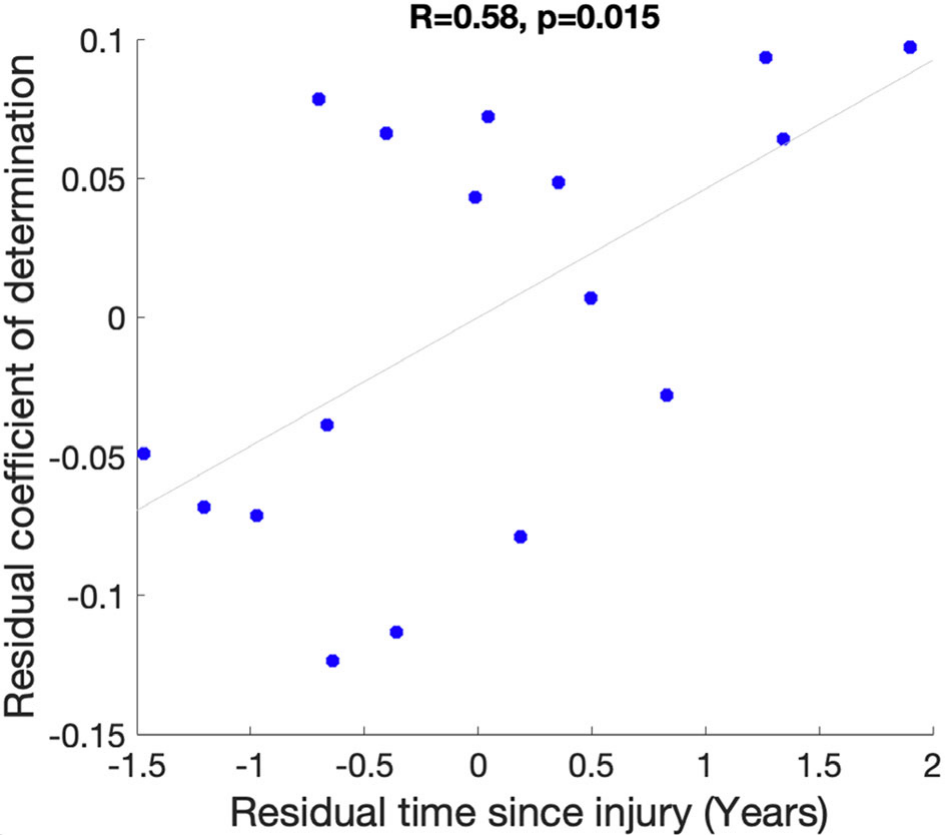
Scatter plot of the relationship between time since injury and coefficient of determination (*r*‐squared) between the measured atrophy and the predicted atrophy. The scatterplot represents the residuals obtained after controlling for the effect of age.

We observed a positive correlation (*R* = 0.58, *P* = 0.015) between time since injury and the coefficient of determination (*R*‐squared) of the association between the measured and predicted injury, after controlling for the effect of age (Fig. 5). There was no significant correlation between time since injury and the model’s peak time (tmax) (*R* = −0.1).

## Discussion

In the present study, we used, for the first time, passive diffusion‐based spread of pathology via the brain’s structural connectome to examine the pattern of neural degeneration in young TBI patients. The model identified subject‐specific epicenters of injury most liable for the distribution of pathology across the brain. Notably, the pattern of degeneration predicted by these injury seeds across individuals comprised principal modes of atrophy with distinct anatomical distribution. These findings demonstrate the potential utility of network spread models in predicting the progression of neural degeneration in future longitudinal studies in TBI patients.

After the initial trauma, the brain undergoes a delayed neurometabolic cascade and white matter degeneration that unfolds over time. This secondary injury is spatially heterogeneous across TBI patients, mainly due to the significant variability in anatomical location of initial injury sites. Here, using a novel automated inference method, we showed that the injury epicenters in the TBI subjects were distributed throughout the temporal (e.g., left superior temporal gyrus, right inferior temporal gyrus, right middle temporal gyrus, left temporal pole), parietal (e.g., inferior parietal gyri), and frontal cortices (e.g., pars orbitalis of the right inferior frontal gyrus), and the striatum (e.g., right caudate nucleus). Importantly, qualitative comparisons of these “epicenter” locations (Data S1) with the sites of injury, using the radiological evidence at the time of injury (Data S1), revealed overlap to some degree in the parietal and temporal regions. In other words, the identified epicenters in these regions may be partially due to multifocal contusive injury from the TBI. Future longitudinal studies are needed to distinguish gray matter thinning from progressive atrophy due to long‐range neurodegenerative processes diffusing along the connectome. In addition, the patterns of degenerative change may be specific to a cohort of young TBI patients and may not generalize to an older sample. Therefore, future studies need to investigate patterns of degenerative change in a sample of adult TBI patients, to examine whether the same pattern would occur if age of initial injury were in adulthood.

Comparison of the predicted patterns with measured atrophy showed moderate‐to‐strong positive associations in all TBI patients. These findings provide support for the ability of the NDM to predict future atrophy patterns in TBI patients. Interestingly, we observed a significant positive correlation between time since injury and the inferred degeneration pattern. However, weak direct relationships were found between time since injury and the model’s peak time. Thus, patients with longer time since injury showed a better correspondence between measured and predicted atrophy patterns. This result indicates that the NDM can capture the pathological progression in the chronic phase of young TBI patients.

An exploratory PCA revealed a spatial structure within the atrophy maps predicted by the injury epicenters. In particular, the hippocampus, parahippocampal gyri, striatum, and temporal cortices were the most prevalent regions among the inferred seeds in our young TBI patients. The patterns of injury epicenters were also consistent with the known vulnerable brain regions in TBI patients. Previous anatomical MRI studies, using either cortical thickness or volumetric measures from regions of interest, have revealed that atrophy of the hippocampus is a widely replicated finding in the chronic phase of moderate‐to‐severe TBI.^30^, ^31^ Our results corroborate previous post‐mortem examinations in individuals with TBI. For example, a post‐mortem study of survivors of a single TBI showed increased neurofibrillary tangle in the cingulate gyrus, superior frontal gyrus, and insular cortex.^32^ In addition, our findings show partial overlap with the spatial pattern of protein deposition as revealed by PET studies in TBI patients. These studies provide support for the accuracy of the proposed inference method. For example, in Mohamed et al. (2019),^33^ elevated tau deposition was found in widespread brain regions, including the cingulate, basal ganglia, temporal pole, superior temporal gyrus, postcentral gyrus, and insula of veterans with TBI compared with controls. In another PET study, Takahata and colleagues (2019)^17^ revealed tau deposits in widespread brain regions, including the temporal gray matter, compared to age‐matched healthy controls. Increased amyloid deposition has been found in the posterior cingulate cortex and striatum in a PET study of Scott and colleagues (2016).^34^ Future studies should correlate the data obtained from PET scans with the epicenters of degeneration revealed by the NDM, to investigate whether regions of toxic protein deposition are consistent with the regions of neurodegenerative spread.

The NDM has a number of advantages. The model predictions can be tested using cross‐sectional data. The model is a quantitative and deterministic assessment tool of spread, moving away from descriptive graph metrics of network alterations in TBI patients. It can handle the between‐patient heterogeneity in the topography of the lesions. It is simple and does not require a lot of computational power. In addition, the method can be applied to any data (z‐scores) that change over time (e.g., white matter microstructure, mean diffusivity, etc.).

Despite these technical advantages, the validity of the NDM depends on the accuracy of the volumetric and tractography processing pipelines. Currently, there is no consensus regarding which weighting factor in the construction of the graphs is the most representative measure of structural connectivity. Other definitions of edge weight, such as fractional anisotropy, mean diffusivity, level of myelination, might also be used in further work.^35^ Another important limitation of the present study is the relatively small sample size. Notwithstanding, the study provides proof‐of‐concept to enable the use of similar modeling techniques in larger groups to confirm and extend our results. In addition, we recognize that variability and heterogeneity are hallmarks of TBI. However, our main analyses were focused on the prediction of inferred degeneration patterns at the individual level. It is important to note that this proof‐of‐concept study used cross‐sectional data and is therefore looking at differences in volume between patients and controls, rather than atrophy per se. Under the assumption that prior to TBI, the brains of all participants were drawn from the same general population, it is a reasonable assumption that such volumetric differences are reflective of atrophy. However, a direct study of atrophy would require a longitudinal experimental design, which is the subject of ongoing work. Specifically, future studies need to identify subject‐specific patterns of neurodegeneration over time using anatomical (T1‐weighted) magnetic resonance imaging (MRI) scans that relate to future spread of disease in patients with and without cognitive deficits TBI.

Aside from these limitations, the present modeling work represents an important contribution to the field of post‐traumatic neurodegeneration because there are few imaging biomarkers that have been developed to track and predict neurodegeneration in the TBI populations. Moreover, using network diffusion modeling, we were able to predict an individual subject’s atrophy pattern and time since injury, highlighting its utility as a promising tool to improve TBI prognosis, including predicting future patterns of atrophy based on patients’ current patterns, identifying young patients with risk of developing an aggressive neurodegenerative disease later in life, and monitoring atrophy patterns in large‐scale clinical trials.

## Conflict of Interest

Govinda Poudel: Nothing to report. Juan F. Dominguez D: Nothing to report. Helena Verhelst: Nothing to report. Catharine Vander Linden: Nothing to report. Karel Deblaere: Nothing to report. Derek Jones: Nothing to report. Ester Cerin: Nothing to report. Guy Vingerhoets: Nothing to report. Karen Caeyenberghs: Nothing to report.

## Authors’ Contributions

Govinda Poudel, PhD: Design and conceptualized study, analyzed the data, and drafted the manuscript for intellectual content; Juan F. Dominguez D, PhD: Drafting and revision of manuscript; Helena Verhelst, PhD: Design and conceptualized study, data collection and analysis, and drafting and revision of manuscript; Catharine Vander Linden, MD, PhD: Data collection and analysis, drafting and revision of manuscript; Karel Deblaere, MD, PhD: Data collection and revision of manuscript; Derek K. Jones, PhD: Drafted the manuscript for intellectual content; Ester Cerin, PhD: Drafting and revision of manuscript; Guy Vingerhoets, PhD: Design and conceptualized study, data collection, and revision of manuscript; Karen Caeyenberghs, PhD: Design and conceptualized study; analyzed the data; and drafted the manuscript for intellectual content.

## Supporting information


**Data S1** **.** Overview of demographic and clinical characteristics of the TBI patients. TSI = Time Since Injury; GCS = Glasgow Coma Scale; LOC = loss of consciousness, DAI = diffuse axonal injury; FL = frontal lobe; TL = temporal lobe; PL = parietal lobe; OL = occipital lobe; C = cerebellum; CC = corpus callosum; GM = gray matter; WM = white matter.
**Data S2** **.** The pseudocode of our novel automated inference method to identify several injury epicentres in each individual TBI patient.
**Data S3** **.** The inferred injury epicentres for each TBI patient. Ctx = cortex; lh = left hemisphere; rh = right hemisphere.

## Data Availability

Anonymized data will be shared by request from any qualified investigator.
