# Supplementary material for: Network diffusion modeling predicts neurodegeneration in traumatic brain injury
Source: Ann Clin Transl Neurol. 2020 Feb 27;7(3):270–9. doi: 10.1002/acn3.50984 (PMC7086000; doi:10.1002/acn3.50984)

**Supplemental Material 1.**

|  | Age/Gender/TSI | Cause accident | GCS/coma | Lesion location/pathology (MRI/CT scan at time of injury) |
| --- | --- | --- | --- | --- |
| 1 | 16.38/M/3.83 | Traffic accident | “stuporous” | Contusion left TL, cerebral edema |
| 2 | 16.70/M/3.15 | Traffic accident | GCS = 3 | DAI FL, TL, OL, (hemorrhagic contusion) left OL, right TL, bilateral cerebellum, CC, external capsule, right globus pallidus, left thalamus, right cerebral peduncle, right mesencephalon |
| 3 | 16.67/M/4.55 | Traffic accident | “subcomatose” | DAI left FL, genu and splenium CC, right mesencephalon |
| 4 | 17.54/M/2.24 | Ski accident | Coma >24h | Subdural contusion bilateral FL, atrophy anterior TL, enlarged ventricles, DAI FL, CC |
| 5 | 15.57/F/3.15 | Traffic accident | Coma of 7 weeks | Subdural hematoma right FL, TL, PL |
| 6 | 14.47/F/1.29 | Traffic accident | GCS = 5 | Subdural hematoma right FL, TL, contusion left TL, DAI left FL, TL, CC, right C |
| 7 | 15.54/M/1.67 | Traffic accident | Coma >24h | Hemorrhagic contusion bilateral FL, right gyrus rectus, DAI, subarachnoid haemorrhage |
| 8 | 17.26/M/1.35 | Ski accident | GCS = 7 | Subdural hematoma left hemisphere |
| 9 | 15.99/M/2.79 | Traffic accident | GCS = 7 | Traumatic axonal injuries (hemorrhagic) bilateral FL (right>left), CC (genu, body, splenium), left OL, left lateral thalamus, left lateral TL, right TL |
| 10 | 11.14/F/1.18 | Traffic accident | N/A | subarachnoid haemorrhage right PL, contrecoup injuries left TL, subgaleal hematoma right PL, extra-axial hemosiderin deposits left TL, atrophy lateral left TL, DAI left C, right WM-GM interface PL/OL |
| 11 | 15.48/M/1.89 | Plane accident | GCS = 3 | DAI and punctiform hemorrhages bilateral FL, brainstem |
| 12 | 17.23/F/2.80 | Traffic accident | N/A | Hemorrhagic foci left FL, right FL, contusion right FL, right TL, subdural hematoma right TL |
| 13 | 12.94/F/1.58 | Traffic accident | GCS = 3 | subarachnoid haemorrhage left FL, interhemispheric, perimesencephalic subarachnoid hemorrhage hematoma left ambient cistern, contusion brainstem |
| 14 | 16.03/F/1.25 | Traffic accident | Coma > 24h | diffuse brain edema, hemorrhagic contusion bilateral FL |
| 15 | 13.74/M/1.37 | Traffic accident | GCS = 3 | DAI right corpus caudate nucleus, posterior/splenium CC, right thalamus, left lentiform nucleus, left posterior limb of the internal capsule, epidural hematoma left PL, TL |
| 16 | 17.46/F/1.92 | Horse accident | GCS = 5 | Subarachnoid hemorrhage right TL, hemorrhagic contusion right FL, right tentorial subdural hemorrhage |
| 17 | 16.88/M/4.05 | Traffic accident | N/A | Hemorrhagic contusion right PL and left FL, multiple contusions TL (right>left), punctiform hemorrhages right cerebral peduncle and right WM TL, subcutaneous hematoma RH. |

**Supplemental Material 2.**

Start

Set y to a vector of atrophy (z-scores) in 82 brain regions

Set t=0:19, α=0.25

Set X_t_ to 82 x 20 matrix of output Run NDM as

x_t =_ e^-α^**^Hτ^**f(0), f(0) is initialised repeatedly for each region

Set R_t_ to matrix of (corr(x_t_,y))

Set R_i_ to a vector of max(R_t_)

Set R_i_(find(arg_max_(R_t_))==19)) to 0

Set R_i_(find(arg_max_(R_t_))==0)) to 0

Set R_i_ < (median of non-zero R_i_) to 0

Set initial condition f_(0)_ to R_i_>0

Run Z_t =_ e^-α^**^Hτ^**f_(0)_

Set t_max_ to argmax(corr(Z_t_,y))

Set I to indices corresponding to R_i_ in descending order

Set C_r_ to a vector size 82

For j from 1 to length (R_i_ >0)

Set f_(0)_(jth element of I) to 1

Run NDM as f(t_max_) _=_ e^-α^**^H^** ^tmax^ f(0)

Set j_th_ element of C_r_ to Corr(f_(tmax),_ y)

end

set Ip to a vector from IR(1) to IR(argmax(Cr))

End

**Supplemental Material 3.**
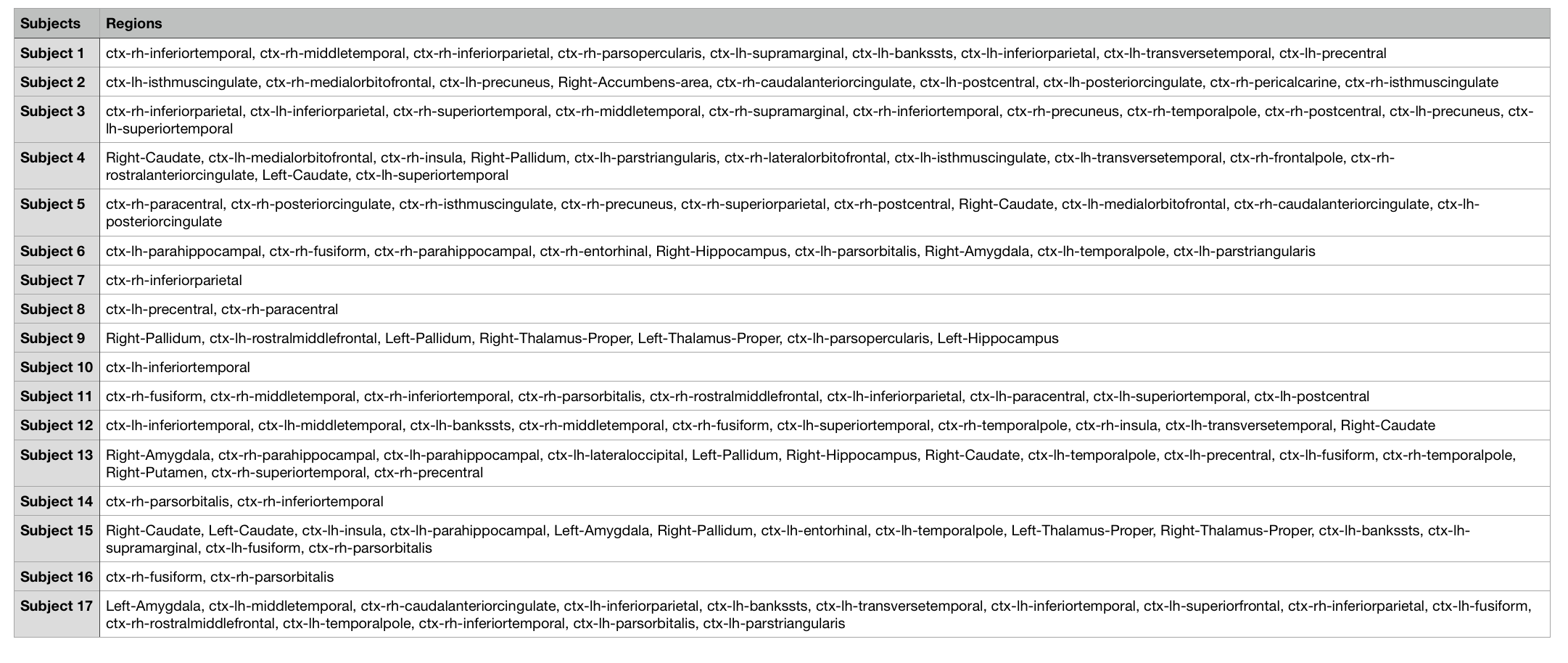

Supplement: Supplementary file 1 — Data S1 . Overview of demographic and clinical characteristics of the TBI patients. TSI = Time Since Injury; GCS = Glasgow Coma Scale; LOC = loss of consciousness, DAI = diffuse axonal injury; FL = frontal lobe; TL = temporal lobe; PL = parietal lobe; OL = occipital lobe; C = cerebellum; CC = corpus callosum; GM = gray matter; WM = white matter. Data S2 . The pseudocode of our novel automated inference method to identify several injury epicentres in each individual TBI patient. Data S3 . The inferred injury epicentres for each TBI patient. Ctx = cortex; lh = left hemisphere; rh = right hemisphere. [file ACN3-7-270-s001.docx]
